# Koch's Notes of Warning

**Published:** 1890-11-29

**Authors:** 


### The Doctor's Armchair.

### KOCH'S NOTES OF WARNING.

When a new theory and method of practice of so important a character as Koch's cure for consumption is put forward, there is always a tendency on the part of critics to take sides. The sanguine man, anxious for great and immediate results, seizes upon facts that encourage his own wishes, and, without taking sufficient time to weigh the evidence, concludes that the positive aspect of the case presents the whole truth. On the other hand, the man who is naturally cautious, or who has been taught caution by disappointing experiences, notes the circumstances that are adverse, the doubtful points in the argument, the weak and sometimes broken links in the chain; and he also, seeing too clearly the defects which his mind has been trained to look for, comes to the conclusion, which he expected from the first to arrive at, and which is the exact opposite of that reached by the man of more sanguine temperament. In the case of Koch and his cure for consumption we have not to do with a problem in metaphysics, for which let us be devoutly thankful! The business is practical, evident to the senses, and may be appreciated by the unlearned as well as the learned. The homely test expressed by the formula—"The proof of the pudding is in the eating"—is the very best which can possibly be applied; and the application of this test in various parts of the world for four or five years will prove to the satisfaction of all men whether Koch is the greatest benefactor of his time, or simply adds one more to the countless number of mistaken medical scientists.

Our attitude in this Journal has been one of criticism. Duty has seemed to us to lie in that direction. But he who appeals to duty must listen to witnesses on both sides, and we desire, therefore, now to call attention to some of Dr. Koch's utterances which go to show that whilst many of his admirers have hoisted the flag of success prematurely, he sees clearly the exact position of affairs, and makes all possible efforts to put other people in possession of his own calm and hopeful, but not too assured state of mind. He does not declare that his cure is one to be applied to all kinds of consumptive cases. On the contrary, he makes it clear that he believes very advanced cases will not be benefited by it at all; that cases in what may be called the middle stage of consumption may or may not improve; and that only cases in the very earliest dawn of the disease may hope to be completely and permanently cured. Not only so, but he takes pains to impress upon medical men the conviction that his cure is not to be looked upon as a supplanter of all other methods of treatment. It is to be considered rather as the leader in the fight, who can only reasonably look for victory when he is properly supported by a well drilled army of capable officers and brave men. On this point Koch says, "I would earnestly warn people against a conventional and indiscriminate application of the remedy in all cases of tuberculosis. The treatment will probably be quite simple in cases where the beginning of phthisis and simple surgical cases are concerned; but in all other forms of tuberculosis medical art must have full sway by careful individualisation, and making use of all other auxiliary methods to assist the action of the remedy. In many cases I had the decided impression that the careful nursing bestowed on the patients had a considerable influence on the result of the treatment, and I am in favour of applying the remedy in proper sanatoria as opposed to treatment at home and in the out-patient room. How far the methods of treatment already recognised as curative—such as mountain climate, fresh air treatment, special diet, &c.—may be profitably combined with the new treatment cannot yet be definitely stated, but I believe that therapeutic methods will also be highly advantageous when combined with the new treatment in many cases, especially in the convalescent stage."

This is the language of common sense, and it might have been much more energetic and decided without making too large an inroad upon medical caution. It is impossible indeed to conceive that any method of treating consumption can be modified otherwise than favourably by abundance of pure air, suitable diet, and perfect hygienic surroundings. All the appliances of this kind that are now at the service of consumptives will still be required, and will, we hope, find exactly what they want in Koch's treatment to make them completely successful. The public, it is to be feared, still run after magic cures, which do their work, nobody knows how, with the promptitude and mystery of a conjuror. The very method adopted by Koch, of simply injecting a little lymph under the skin and awaiting the result, seems to have something of legerdemain about it, and catches the public fancy accordingly. This, which is one of its chief fascinations to the uninstructed, is the very thing upon which the intelligent medical mind fastens with the keenest criticism.

Early diagnosis and prompt treatment: these are the notes of Koch's mind on the new cure. He says: "The most important point to be observed in the new treatment is its early application. The proper subjects for treatment are patients in the initial stage of phthisis, for in them the curative action can be most fully shown, and for this reason, too, it cannot be too seriously pointed out that practitioners must in future be more than ever alive to the importance of diagnosing phthisis in as early a stage as possible. . . . A doctor who shall neglect to diagnose phthisis in its earliest stage by all methods at his command, will be guilty of the most serious neglect of his patient, whose life may depend on this diagnosis, and the specific treatment at once applied in consequence thereof. In doubtful cases medical practitioners must make sure of the presence or absence of tuberculosis, and then only the therapeutic method will become a blessing to suffering humanity when all cases of tuberculosis are treated in their earliest stage, and we no longer meet with neglected serious cases forming an inextinguishable force of fresh infections."

It is only necessary to remark on the subject of early diagnosis that this is emphatically necessary, whether Koch's method be found a veritable cure or not. All practical physicians are agreed that cases of advanced consumption cannot be restored to health either by Koch's treatment or any other. The only hope for consumptives has hitherto lain in early diagnosis and treatment. The great boon and blessing which will come to mankind if Koch's anticipations be realised will be that what has hitherto been only a hope will be converted into a certainty. It is not, however, medical men alone who are responsible for early diagnosis. Patients themselves must take their share of the responsibility. When they find themselves suffering from obscure ailments which debilitate and make them uncomfortable without producing any decided symptoms, they must seek competent medical aid with intelligent promptitude. If medicine is to do all that is possible for men, they must in their turn learn to understand it more intelligently, and to enter into more intimate relations with it.

